# Reply to: Pitfalls in the location of guest molecules in metal-organic frameworks

**DOI:** 10.1038/s41467-022-32891-z

**Published:** 2022-09-09

**Authors:** Bin Wang, Lin-Hua Xie, Daqiang Yuan, Banglin Chen

**Affiliations:** 1 Beijing Key Laboratory for Green Catalysis and Separation and Department of Chemistry and Chemical Engineering, College of Environmental and Energy Engineering, Beijing University of Technology, Beijing, PR China; 2 State Key Laboratory of Structural Chemistry, Fujian Institute of Research on the Structure of Matter, Chinese Academy of Sciences, Fuzhou, PR China; 3 Department of Chemistry, University of Texas at San Antonio, San Antonio, TX USA

**Keywords:** Metal-organic frameworks, Structural properties, Metal-organic frameworks

**replying to** T. Poręba et al. *Nature Communications* 10.1038/s41467-022-32890-0 (2022)

In our published work^1^, we designed and synthesized a stable zirconium-based metal-organic framework named BUT-17, with appropriate one-dimensional hexagonal channels and aromatic-rich pore surfaces for the sensitive and selective sensing of two representative polychlorinated dibenzo-*p*-dioxins (PCDDs), 2,3-dichlorodibenzo-*p*-dioxin (BCDD) and 2,3,7,8-tetrachlorodibenzo-*p*-dioxin (TCDD), based on the fluorescence quenching. The detection limits of BUT-17 toward BCDD and TCDD are as low as 27 and 57 ppb. The sensing mechanism was ascribed to the formation of the nonfluorescent ground-state complexes between the PCDD molecules and the fluorophore of BUT-17, which was supported by the single-crystal structure of BCDD-loaded BUT-17 (BCDD@BUT-17) and adsorption experiments.

In the accompanying comment, Poręba et al. raised concern regarding the single-crystal X-ray diffraction (SCXRD) data of BCDD@BUT-17 in our work. After analyzing the SCXRD data, they believe that the quality of the diffraction data does not allow to reliably determine the presence of BCDD in the pores of BUT-17. Here, we want to emphasize that determining the guests in the pores of MOFs is still a big challenge^2^. Some microporous MOFs have stronger pore confinement effect on trapped small gas or organic molecules, and these confined guests can be possibly determined through SCXRD^3,4^. However, when we move forward determining the structure of guests in MOFs with large-pore sizes, many threats raise. For example, when preparing guest-loaded single crystals using the crystal sponge method (CSM)^5^, it is difficult for the guests to form a fully occupied periodic arrangement in the pores of MOFs due to the facts such as diffusion time and competitive adsorption of solvents. Besides, the pore confinement effect of large-pore MOFs on guest molecules is relatively weaker. Weak host–guest interactions commonly lead to partial disorder or even complete random distribution of guest molecules. Determination of guest molecules in pores sometimes gets more challenging when there exists symmetry-imposed disorder as the host frameworks of many MOFs are in high-symmetry space groups, as in our case. Of course, these difficulties may be eventually overcome with the continuous development of advanced X-ray (or other radiations) diffraction technology.

The single crystals of BCDD@BUT-17 were obtained using the CSM. After immersing in a hexane solution of BCDD (1000 ppm) for 48 h, a single crystal of BCDD@BUT-17 was picked up and mounted on a single-crystal X-ray diffractometer and heated under the N_2_ flow (50 °C) for 5 h to remove the residuary solvent in the pores of BUT-17. After that, a set of diffraction data (129,085 reflections, *R*_int_ = 0.1145) was collected at 100 K. According to systematic absences, the space group was determined to be *P*6_3_/*mmc* (No. 194). The statistics vs. resolution table is shown Table S1. As the single crystal was subject to heating at 150 °C and BCDD loading, the dataset was regarded to be acceptable, although not in high quality.

The host framework of BCDD@BUT-17 was successfully solved with the SHELXT program. After refinements of all the framework non-hydrogen atoms with anisotropic thermal parameters, we carefully checked the *F*_obs_ − *F*_calc_ electron density map. It was observed that several weak Q-peaks inside the voids formed a model like a disordered large planer molecule, which located around the surface of the large hexagonal pores (Supplementary Fig. 1). Considering that (1) the hexane solution of BCDD where the guest-free single crystal of BUT-17 was immersed was in a low concentration (1000 ppm) (the loading of BCDD in BUT-17 could be quite low), (2) the hexagonal pores in BUT-17 are as large as 24 Å in diameter (the guest molecules were likely highly ordered), and (3) BUT-17 could remove BCDD in its acetone aqueous solution with a removing efficiency of 73% (BCDD could be adsorbed by BUT-17), this model was assigned to be a guest BCDD molecule although the electron densities of these Q-peaks were relatively low (0.76 to 1.20 e/Å^3^). Restraint instructions (SADI, DFIX, FLAT, DELU, and SIMU) were applied for the following refinements. Such restraint instructions are commonly used in the refinements of disordered structures. The 2D (0.20–1.65 e/Å^3^) and 3D (0.50 e/Å^3^) *F*_obs_ electron density maps also indicated the diffuse distribution of the guest BCDD molecule (Fig. 1a, b).

**Fig. 1:**
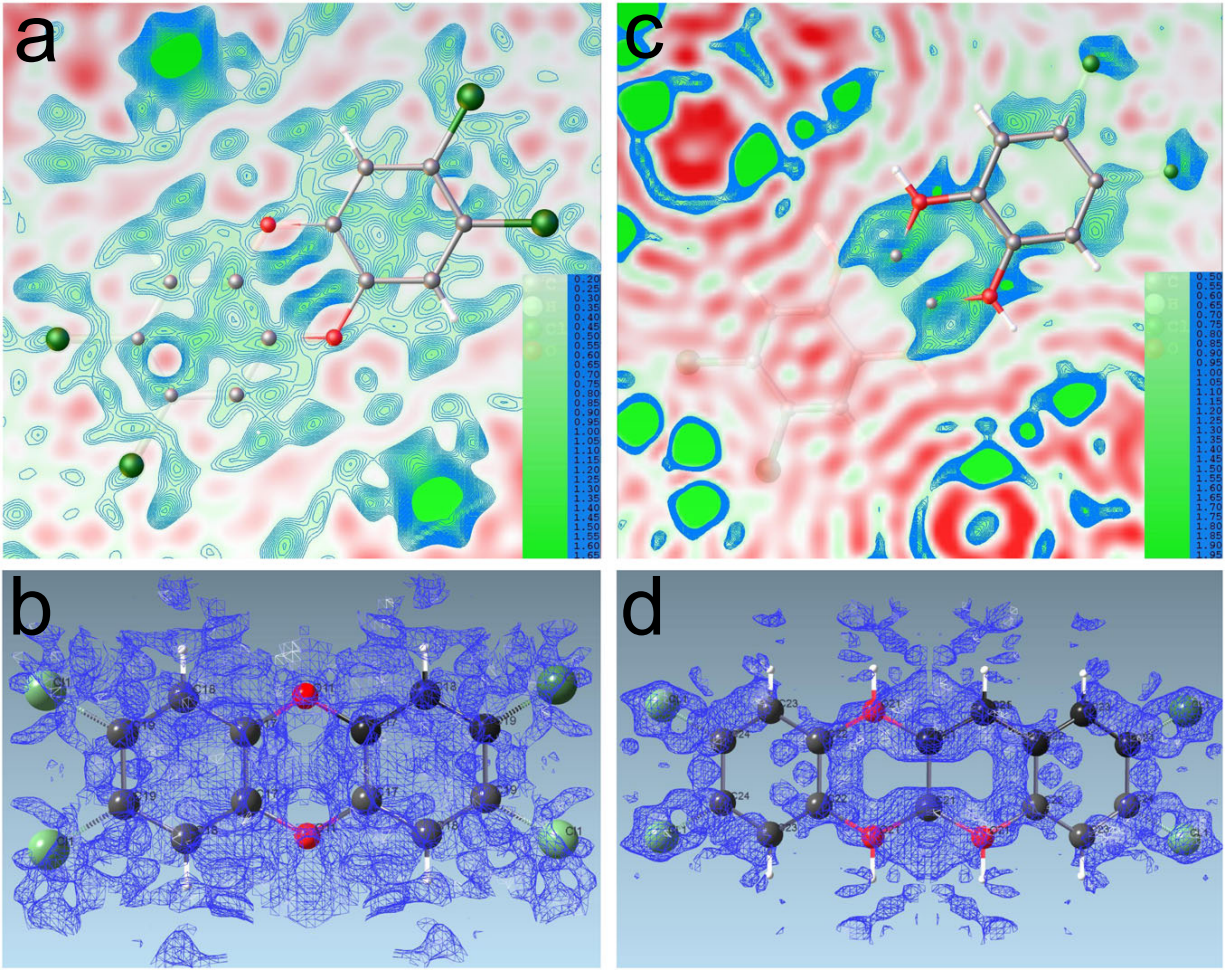
*F*_obs_ electron density maps of BCDD molecules in BUT-17. The 2D and 3D *F*_obs_ electron density maps for the BCDD molecules observed by the previously reported (**a**, **b**) and newly collected SCXRD data (**c**, **d**) of BCDD@BUT-17, respectively.

To clarify the concern from Poręba et al. we collected a new set of SCXRD data for BCDD@BUT-17 by treating the BUT-17 crystals under a milder condition (see supporting inforamtion) and using a more advanced single-crystal X-ray diffractometer (XtaLAB Synergy-i diffractometer). As shown in Supplementary Table 2, the data were largely improved. A clear model of BCDD was found in the *F*_obs_ − *F*_calc_ electron density map at the same location in BUT-17, which was disordered at two positions with a slightly bent configuration (Supplementary Fig. 2). Stronger electron density of the BCDD molecule could be found in the 2D (0.50–1.95 e/Å^3^) and 3D (0.75 e/Å^3^) *F*_obs_ maps (Fig. 1c, d). Final refinements with a occupancy of 30% for the BCDD molecule gave satisfactory agreement factors (*R*_1_ = 0.0463, *wR*_2_ = 0.1352, Fig. 2 and Supplementary Table 3). The new SCXRD data justify our previous assignment of guest BCDD in the pore of BUT-17. To further confirm that BCDD was absorbed by BUT-17, ^1^H NMR spectrum of the digested BCDD@BUT-17 was measured (see supporting inforamtion). As shown in Supplementary Fig. 4, the molar ratio of ligand to BCDD in BCDD@BUT-17 was estimated to be 1/0.16, consistent with that obtain from the single-crystal structure (1/0.15), further validating the rationality of the obtained single-crystal structures of BCDD@BUT-17.

**Fig. 2:**
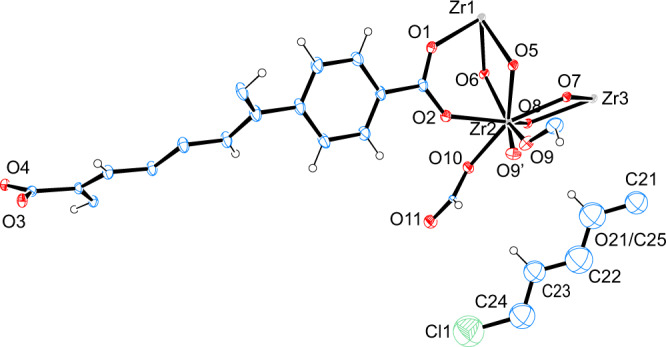
The asymmetry unit of BCDD@BUT-17. ORTEP view for the asymmetry unit of BCDD@BUT-17 determined from the newly collected SCXRD data. Displacement ellipsoids are represented by 30% probability level.

In conclusion, we thank Dr. Poręba for raising this issue. From the crystallography point of view, we admit that the diffraction data provided in our article was not good enough to clearly show that BCDD was indeed absorbed into the pores of BUT-17. Based on a set of better diffraction data and ^1^H NMR spectra analyses, we were able to get stronger evidence of the presence of BCDD in BUT-17. We hope this discussion could provide positive insights into the future structural studies of guest molecules in large-pore MOFs.

## Supplementary information

Supplementary Information

## Data Availability

Crystallographic data for the new collected structure of BUT-17@BCDD has been deposited at the Cambridge Crystallographic Data Center, under deposition number CCDC 2192596. Copies of the data can be obtained free of charge via www.ccdc.cam.ac.uk/data_request/cif. All other data supporting the findings of this study are available within the article and its Supplementary Information, or from the corresponding author upon reasonable request.
