# Supplementary material for: Reply to: Pitfalls in the location of guest molecules in metal-organic frameworks
Source: Nat Commun. 2022 Sep 9;13:5287. doi: 10.1038/s41467-022-32891-z (PMC9463437; doi:10.1038/s41467-022-32891-z)
Supplement: Supplementary file 1 — Supplementary Information [file 41467_2022_32891_MOESM1_ESM.pdf]

Reply to: Pitfalls in the location of guest molecules in metal-organic frameworks

Wang et al.

Supplementary Information

Supplementary Methods

Preparation of single crystals of BCDD@BUT-17 under a milder condition. As-synthesized single crystals of BUT-17 were washed with fresh DMF several times to remove the unreacted reactants and residual dimethylamine. Fresh acetone was subsequently added, and the sample was allowed to stay in it for 8 h. This procedure was repeated three times over one day. After washed with dry hexane for several times, the single crystals of BUT-17 were immersed in the hexane solution of BCDD (1000 ppm) for 48 h. After that, one single crystal of BCDD@BUT-17 was picked up and placed on the single-crystal X-ray diffractometer. A new set of diffraction data at 100 K was collected for BCDD@BUT-17.

Single-crystal X-ray diffraction. The crystal data of BCDD@BUT-17 were collected on a XtaLAB Synergy-i diffractometer equipped with a graphite-monochromatic enhanced Cu Kα radiation (λ = 1.54184 Å) at 100 K. The datasets were corrected by empirical absorption correction using spherical harmonics, implemented in the *SCALE3 ABSPACk* scaling algorithm. The structure of BCDD@BUT-17 was solved by direct methods and refined by full-matrix least-squares on F^2 with anisotropic displacement using the *SHELXTL* software package. Non-hydrogen atoms were refined with anisotropic displacement parameters during the final cycles. Hydrogen atoms of the ligands were calculated in ideal positions with isotropic displacement parameters. After determination of the guest BCDD molecules, there are still large solvent accessible pore volume in the pore of BUT-17, which should be occupied by highly disordered solvent molecules. No satisfactory structural models for these highly disordered solvent molecules could be assigned, and therefore the *SQUEEZE* program implemented in *PLATON* was used to remove the electron densities of these disordered species. The details of structural refinement can be found in Supplementary Table 3.

^{1}H NMR measurements. The single crystals of BCDD@BUT-17 immersed in the hexane solution of BCDD for 48 h were centrifuged and thoroughly washed with hexane (3 times) and dried in a vacuum oven under 80 ^oC for 6 h. Then, the activated BCDD@BUT-17 was placed in an NMR tube, and a mixture of DCl (20 µL) and DMSO-d_6 (500 µL) was added. The NMR tube was subsequently heated in an oil bath at 120 ^oC for 5 h. Then, the ^{1}H NMR spectrum of the dissolved BCDD@BUT-17 was recorded. As shown in Supplementary Figure 3, we can easily find the peaks of BCDD molecules in the ^{1}H NMR spectrum of digested BCDD@BUT-17, undisputedly indicating the existence of BCDD molecules in the pores of BUT-17. It should be

noted that, under such conditions, part single crystals still existed, which means that BUT-17 was not fully digested. In the meantime, however, we believe that the absorbed guests were totally dissolved. Thus, the ligand/BCDD molar ratio estimated from the ^{1}H NMR spectrum did not reflect the actual ratio in the single crystals. Under similar conditions and using a powder sample of the MOF (smaller crystal size), we were able to obtain a ^{1}H NMR spectrum of totally digested BCDD@BUT-17, which showed a ligand/BCDD molar ratio of 1:0.16 (Supplementary Figure 4), which was consistent with that obtain from the newly collected single-crystal structure of BCDD@BUT-17 (1:0.15).

Supplementary Table 1. The intensity statistics for reported dataset of BCDD@BUT-17

Resolution	#Data	#Theory	%Complete	Redundancy	Mean I	Mean I/s	R(int)	R(sigma)
Inf-2.30	480	487	98.6	16.19	224.8	42.47	0.0469	0.0134
2.30-1.75	544	544	100	16.77	172.3	26.96	0.0579	0.0215
1.75-1.50	550	550	100	14.74	146.9	20.18	0.0703	0.0308
1.50-1.35	556	556	100	20.82	76.9	17.09	0.1134	0.0382
1.35-1.25	520	520	100	24.73	32.7	11.9	0.1967	0.0558
1.25-1.15	707	707	100	23.13	41.6	12.65	0.1913	0.0508
1.15-1.05	1008	1008	100	13.7	51.7	12.12	0.1471	0.0543
1.05-1.00	643	643	100	12.27	39.2	9.9	0.1614	0.067
1.00-0.95	805	805	100	11.61	30.6	8.28	0.1947	0.0801
0.95-0.90	975	975	100	10.83	26.2	8.2	0.212	0.088
0.90-0.85	1219	1219	100	9.63	20.5	7.32	0.2507	0.1014
0.85-0.80	1509	1540	98	6.39	16.4	6.27	0.2597	0.1223
0.80-0.80	5	40	12.5	0.2	4.9	2.08	0.0738	0.4204
0.90-0.80	2733	2799	97.6	7.71	18.2	6.73	0.2542	0.112
Inf-0.80	9521	9594	99.2	13.45	57.6	12.86	0.1145	0.0443

Supplementary Table 2. The intensity statistics for newly collected dataset of BCDD@BUT-17

Resolution	#Data	#Theory	%Complete	Redundancy	Mean I	Mean I/s	R(int)	R(sigma)
Inf-2.30	487	489	99.6	6.11	352.6	51.33	0.0265	0.0179
2.30-1.80	467	467	100	11.22	241.3	62.6	0.0306	0.013
1.80-1.55	496	496	100	17.11	228.6	76.33	0.0315	0.0099
1.55-1.40	482	482	100	19.2	172.6	78.87	0.0327	0.0097
1.40-1.25	730	730	100	18.48	74.9	57.88	0.0426	0.0121
1.25-1.15	718	718	100	17.39	82.5	54.34	0.0468	0.0125
1.15-1.05	997	997	100	15.83	94.2	54.03	0.0454	0.0126

1.05-1.00	665	665	100	11.44	79	45.43	0.0434	0.015
1.00-0.95	799	799	100	10.36	59.5	38.77	0.0543	0.017
0.95-0.90	990	990	100	9.2	53.6	37.57	0.0709	0.0193
0.90-0.85	1221	1221	100	7.61	43.8	34.57	0.0592	0.02
0.85-0.80	1173	1549	75.7	2.85	36.8	22.88	0.0449	0.0306
0.80-0.80	41	120	34.2	0.47	35.8	17.06	0.0388	0.0432
0.90-0.80	2435	2890	84.3	4.76	40.3	28.64	0.0551	0.025
Inf-0.80	9266	9723	95.3	10.95	101.5	46.77	0.0401	0.015

Supplementary Table 3. Crystal data and structure refinement results for BCDD@BUT-17 (newly collected)

Empirical formula	$C_{62.60}H_{32.20}Cl_{0.60}O_{34.60}Zr_6$
Formula weight	1906.47
Temperature	99.98(12) K
Wavelength	1.54178 Å
Crystal system	Hexagonal
Space group	$P6_3/mmc$
Unit cell dimensions	a = 32.72780(10) Å
	c = 27.28710(10) Å
Volume	25311.71(18) Å3
Z	6
Density (calculated)	0.750 Mg/m^3
Absorption coefficient	3.359 mm^{-1}
F(000)	5609
Theta range for data collection	3.118 to 75.486°
Index ranges	-38<=h<=39, -37<=k<=38, -34<=l<=12
Reflections collected	104501
Independent reflections	8976 (R_{int} = 0.0399)
Completeness to theta = 67.679°	99.8 %
Absorption correction	Semi-empirical from equivalents
Max. and min. transmission	1.00000 and 0.63628
Refinement method	Full-matrix least-squares on F^2
Data / restraints / parameters	8976 / 94 / 274
Goodness-of-fit on F^2	1.055
Final R indices [I > 2sigma(I)]	$R_1{}^a$ = 0.0422, $wR_2{}^b$ = 0.1301
R indices (all data)	R_1 = 0.0463, wR_2 = 0.1352
Largest diff. peak and hole	1.908 and -0.840 e.Å^{-3}

^{a} $R_1 = \sum||F_o| - |F_c||/\sum|F_o|$. ^{b} $wR_2 = [\sum w(F_o{}^2 - F_c{}^2)^2/\sum w(F_o{}^2)^2]^{1/2}$

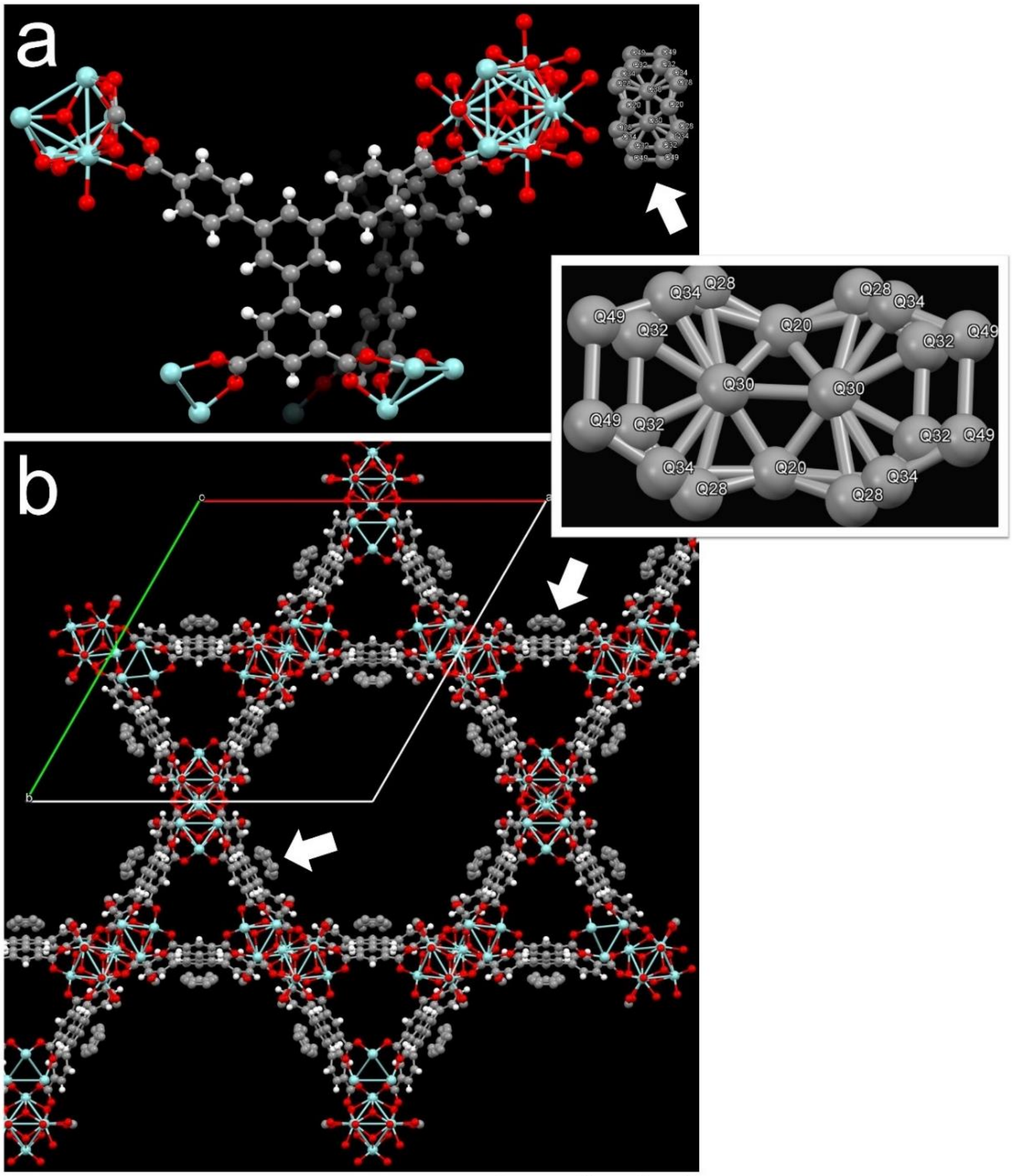

		x	y	z	sof	U	Peak
Q20	1	0.2393	-0.1802	0.2500	10.50000	0.05	1.20
Q28	1	0.2475	-0.1622	0.2870	11.00000	0.05	0.99
Q30	1	0.7969	0.2031	0.7238	10.50000	0.05	0.98
Q32	1	0.8188	0.2203	0.6821	11.00000	0.05	0.96
Q34	1	0.2381	-0.1656	0.3032	11.00000	0.05	0.95
Q49	1	0.8255	0.2174	0.6613	11.00000	0.05	0.76

Supplementary Figure 1. Modeling of guest molecules in single-crystal structure refinements. **a** Initial model of the guest BCDD molecule found in the difference Fouier map (F_o-F_c) for the reported SCXRD data of BCDD@BUT-17, **b** Perspective view of the guest BCDD molecule locating at the sites near the channel walls.

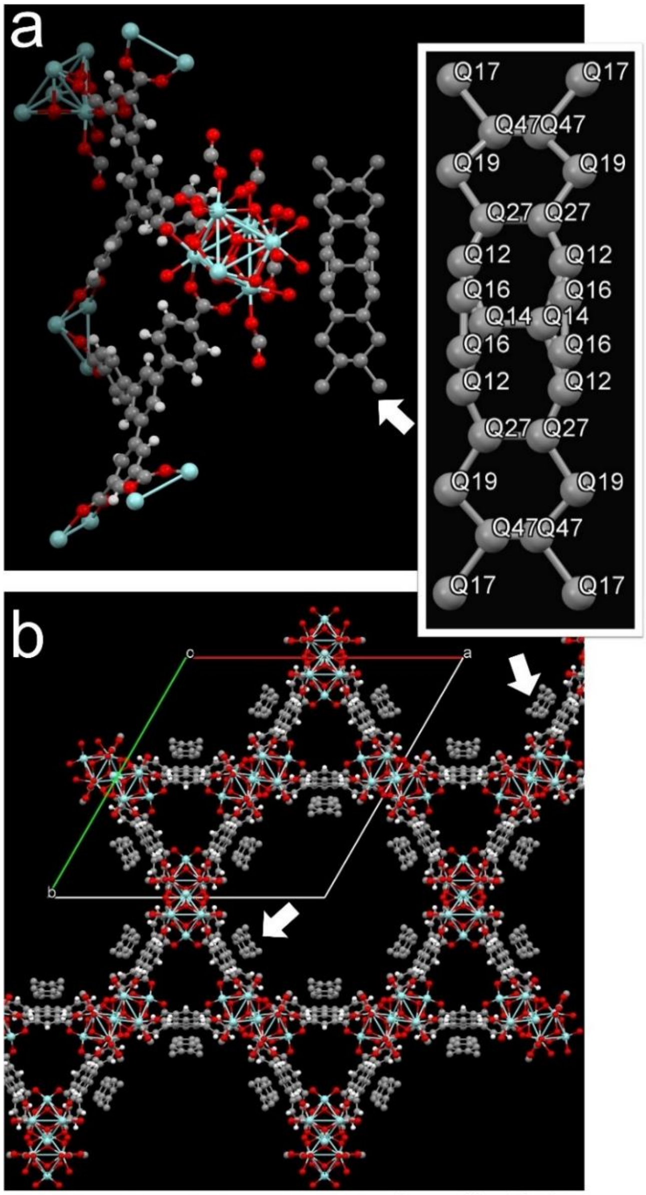

		x	y	z	sof	U	Peak
Q12	1	0.5992	-0.1618	0.3087	11.00000	0.05	1.77
Q14	1	0.5904	-0.1830	0.2500	10.50000	0.05	1.66
Q16	1	0.5948	-0.1642	0.2753	11.00000	0.05	1.57
Q17	1	0.7768	0.1253	0.5118	11.00000	0.05	1.56
Q19	1	0.8588	0.2370	0.6023	11.00000	0.05	1.45
Q27	1	0.8271	0.2168	0.6500	11.00000	0.05	1.13
Q47	1	0.8027	0.1635	0.5629	11.00000	0.05	0.75

Supplementary Figure 2. Modeling of guest molecules in single-crystal structure refinements. **a** Initial model of the guest BCDD molecule found in the difference Fouier map (F_o-F_c) for the newly collected SCXRD data of BCDD@BUT-17, **b** Perspective view of the guest BCDD molecule locating at the sites near the channel walls.

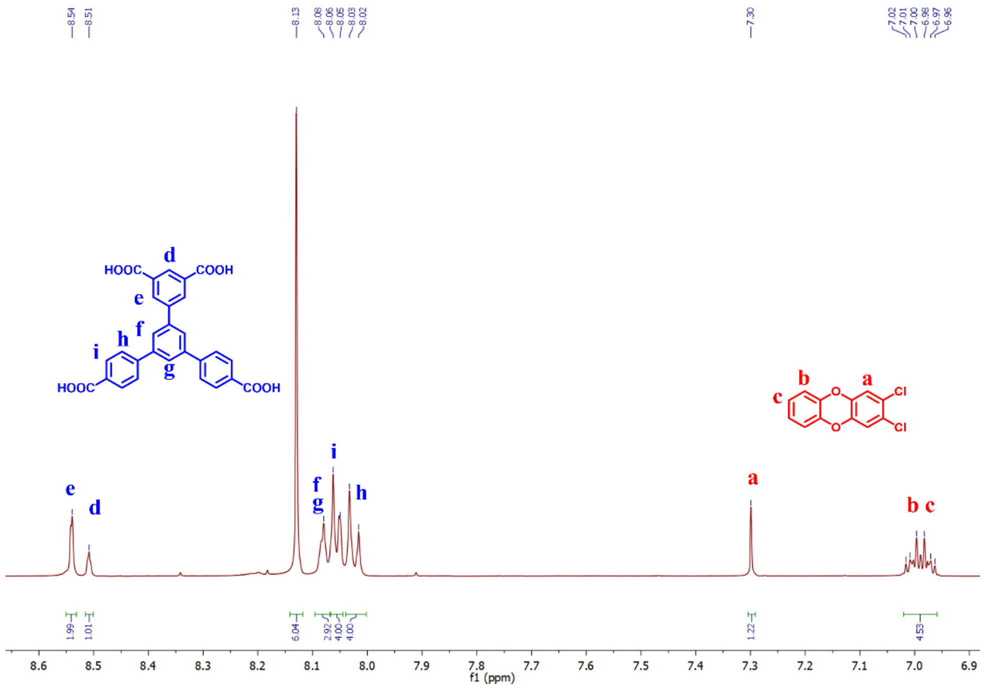

Supplementary Figure 3. Liquid NMR spectrum. ^{1}H NMR spectrum of the partial digested single crystals of BCDD@BUT-17.

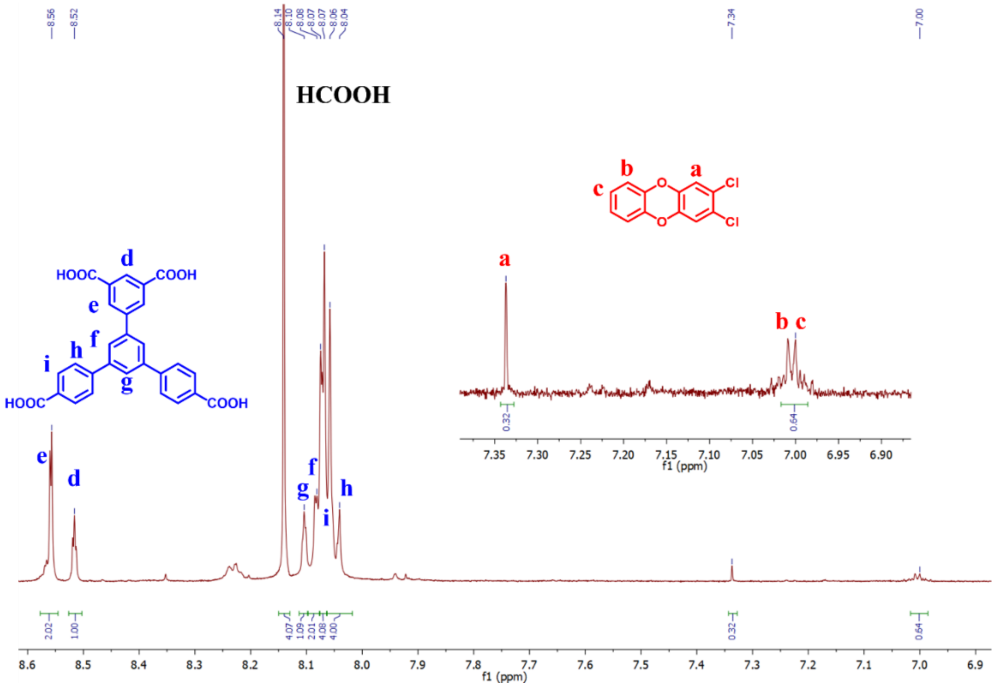

Supplementary Figure 4. Liquid NMR spectrum. ^{1}H NMR spectrum of the fully digested powder of BCDD@BUT-17.
